# Explaining the universality of biological thermal responses

**DOI:** 10.1073/pnas.2610187123

**Published:** 2026-05-26

**Authors:** Jose Ignacio Arroyo, Christopher Kempes, Geoffrey West, Pablo A. Marquet

**Affiliations:** ^a^Santa Fe Institute, Santa Fe, NM; ^b^Centro de Modelamiento Matemático (CMM), Universidad de Chile, Santiago 8370459, Chile; ^c^Facultad de Ciencias Biológicas, Pontificia Universidad Católica de Chile, Santiago 8331150, Chile; ^d^Instituto de Sistemas Complejos de Valparaíso, Valparaíso 2360155, Chile; ^e^Wissenschaftskolleg zu Berlin, Berlin 14193, Germany

Arnoldi et al. (1) developed an approximated phenomenological mathematical method to collapse data of thermal performance curves (TPCs) into a left-skewed dimensionless universal curve, and applied it successfully to two different phenomenological models, but their method failed to be applied to a first-principles-based model developed by us (2, 3), proving that their method is not general.

Universality refers to the property that many physical systems exhibit the same macroscopic quantitative properties despite having microscopic differences (4). Data collapse through a dimensionless equation is one aspect of demonstrating universality. Commonly, to derive a dimensionless equation, the original equation is transformed so that the dependent and independent variables have no units. Such a dimensionless expression can be derived by dividing both sides of an equation by its value at a given point (e.g., an inflection point) (5). Arnoldi et al. do this, but in addition, they used a Taylor expansion on one of the terms of the equation; their formula is an approximation. However, beyond data collapse through a dimensionless equation, it is also helpful to have a theory based on first principles that explains the mechanisms by which the phenomenon occurs in different systems or scales, something that Arnoldi et al. do not do.

Arnoldi et al. argue that a “complete explanation for the universality of left-skewed TPCs remains elusive.” However, this is misleading, since not all temperature response patterns are concave and left-skewed (3, 6, 7), hence left-skewed TPCs are not universal. There are examples of variables that exhibit approximately symmetrical or right-skewed responses for concave curves (3, 6, 7), and there are also convex curves (2). The only property that temperature responses have in common, from the enzymatic to the socioeconomic level, is that they have a critical point. In this regard, Arroyo et al. (2) derived a minimal model that establishes the principles that explain this criticality and can account relatively well for all functional forms seen in thermal responses, including concave, convex, asymmetric -both left- and right-skewed, and approximately symmetrical ones.

Arnoldi et al. report that two models—except ours—converge to their formulation, and interpret this as evidence against our approach. If anything, we take it as evidence that our approach is different from the other two models, and it is more general and epistemologically more robust, as it is based on an accepted theory for chemical reaction kinetics based on the first principles of quantum mechanics that we expanded to the macroscopic level. They argue that reducing our model to theirs yields a Gaussian curve that lacks the characteristic left skew. However, this conclusion is problematic. First, data collapse is not predicated on preserving the same functional form of the original noncollapsed data; this can change (5). Second, the dimensionless form derived in Arroyo et al. is not Gaussian; rather, the Gaussian shape emerges only after the transformations and approximations introduced by Arnoldi et al. Thus, the resulting curve reflects their methodological choices, not an inherent property of our model. Further, it is not clear how they derive such a Gaussian-like expression.
